# Editorial: Epigenetics and metabolism

**DOI:** 10.3389/fendo.2024.1373368

**Published:** 2024-02-09

**Authors:** Nico Mitro, Francisco Verdeguer, Valentina Perissi

**Affiliations:** ^1^ DiSFeB, Dipartimento di Scienze Farmacologiche e Biomolecolari “Rodolfo Paoletti”, University of Milan, Milan, Italy; ^2^ Department of Experimental Oncology, European Institute of Oncology (IEO), IRCCS, Milan, Italy; ^3^ InSphero AG, Schlieren, Switzerland; ^4^ Biochemistry&Cell Biology Department, Chobanian&Avedisian School of Medicine, Boston University, Boston, MA, United States

**Keywords:** nucleotide, adipose tissue, lysine and DNA methylation, ADP ribosylation, acetyl-CoA producing enzymes, epigenetics, metabolism

In the intricate coordination of cellular processes, the interplay between metabolism and epigenetics has emerged as an intriguing frontier, revealing a previously overlooked layer of regulation in the complex choreography of molecular mechanisms. While metabolism has traditionally been seen as the driving force behind cellular energy production, and epigenetics as the conductor orchestrating the symphony of gene expression, they are now substantially acknowledged as interlinked collaborators that shape the fate of cells.

The metabolic pathways linked to cellular respiration, nutrient utilization, and biosynthesis are now recognized as key regulators influencing the epigenome. The epigenome, a dynamic set of chemical modifications to DNA and histone proteins, dictates the accessibility of genes and plays a pivotal role in determining cellular identity, function, and fate. Understanding how these two seemingly disparate fields intersect is crucial for unraveling the complex language that governs cellular behavior in physiological and pathological settings.

This introduction lays the groundwork for a thorough exploration of the profound connections between metabolism and epigenetics, drawing inspiration from recent research that has brought to light this complex relationship. This research has delved deep into the molecular events governing nucleotide metabolism, adipose tissue heterogeneity, histone lysine methylation, and the epigenetics of diabetic cardiomyopathy. Additionally, studies have explored the cooperative targeting of Poly (ADP-Ribose) Polymerase 1 (PARP-1) domains and the role of acetyl-CoA producing enzymes in shaping the epigenetic landscape.

This editorial seeks to weave together the insights from six noteworthy articles published in Frontiers in Endocrinology in the topic section “Epigenetics and Metabolism”, providing a comprehensive understanding of the latest advancements in this field.

The first article by Suganuma and Workman explores the complex relationship between nucleotide metabolism and epigenetics. Nucleotides, the building blocks of DNA and RNA, are integral to cellular function and replication. The authors delve into the role of nucleotide metabolism in shaping epigenetic modifications, shedding light on the interconnectedness of these fundamental cellular processes. Understanding this nexus is crucial for unraveling the mechanisms underlying gene expression and cellular differentiation.

Adipose tissue, once considered a mere energy reservoir, is now recognized as a dynamic organ with heterogenic cell populations. By deconstructing adipose tissue at the single-cell level, Duerre and Galmozzi reveal the complexity of its cellular composition. This newfound understanding opens avenues for targeted interventions in metabolic disorders, providing a nuanced perspective on the role of adipose tissue in health and disease.

Moving into the cardiovascular field, Cao et al. explore the role of histone lysine methylation in vascular calcification. Vascular calcification, a hallmark of cardiovascular diseases, involves the deposition of minerals in blood vessel walls. The authors elucidate how histone lysine methylation modifications contribute to the regulation of genes involved in this pathological process. This research provides crucial insights into potential epigenetic targets for mitigating vascular calcification and associated cardiovascular risks.

Methylation modifications of DNA play a pivotal role in the context of the epigenetic landscape of diabetic cardiomyopathy, a serious complication of diabetes affecting the heart. By unraveling the epigenetic modifications associated with diabetic cardiomyopathy, Hao and Liu pave the way for developing targeted therapeutic strategies to mitigate the impact of diabetes on cardiac function.

Bamgbose et al. delve into the cooperative targeting of PARP-1 domains, shedding light on their role in regulating metabolic and developmental genes. PARP-1, a key player in DNA repair, emerges as a multifaceted regulator with implications beyond its traditional role. Understanding the cooperative targeting mechanisms provides novel insights into the crosstalk between DNA repair processes and the regulation of essential cellular functions.

Russo et al. contribute to the discussion by providing novel insights into the role of acetyl-CoA producing enzymes in epigenetic regulation. Acetyl-CoA is a central metabolite linking cellular metabolism to epigenetic modifications. The authors illuminate how enzymes involved in acetyl-CoA production influence chromatin dynamics and gene expression, highlighting the intricate interplay between cellular metabolism and epigenetic regulation.

In summarizing these diverse threads of research, a cohesive narrative emerges, highlighting the sophisticated network of interactions that govern endocrine processes. From the fundamental role of nucleotide metabolism to the nuanced heterogeneity of adipose tissue, and from the epigenetic signatures of vascular calcification to the complexities of diabetic cardiomyopathy, researchers are steadily deciphering the language written in the molecular code. The cooperative targeting of PARP-1 domains and the role of acetyl-CoA producing enzymes add additional layers to this narrative, emphasizing the interconnectedness of cellular processes.

As we stand at the intersection of metabolism and epigenetics, these findings not only deepen our understanding of fundamental biological processes but also offer promising avenues for therapeutic interventions. The road ahead involves further exploration of these key molecular mechanisms, pushing the boundaries of our knowledge and translating these discoveries into innovative medical strategies. The tapestry of endocrinology is rich and complex, and as each new study adds a thread, the picture becomes clearer, bringing us closer to unlocking the secrets of cellular regulation and disease.

## Author contributions

NM: Writing – original draft, Writing – review & editing. FV: Writing – review & editing. VP: Writing – review & editing.

